# Special Section Guest Editorial: Seeing Inside Tissue with Optical Molecular Probes

**DOI:** 10.1117/1.JBO.28.8.082801

**Published:** 2023-08-30

**Authors:** Nada N. Boustany, Mark Niedre, Manojit Pramanik

**Affiliations:** a Rutgers University, Department of Biomedical Engineering, Piscataway, New Jersey, United States; b Northeastern University, Department of Bioengineering, Boston, Massachusetts, United States; c Iowa State University, Department of Electrical & Computer Engineering, Ames, Iowa, United States

## Abstract

The editorial introduces the Special Section on Seeing Inside Tissue with Optical Molecular Probes.

Although fluorescence contrast has been used in medical imaging and pre-clinical research contexts for decades, the last 10 years have seen rapid growth in the development of molecularly specific optical contrast agents. Whereas the first fluorophores used for biomedical applications—such as methylene blue and indocyanine green (ICG)—were generally non-targeted and exhibited sub-optimal photochemical and photobiological properties, newer contrast agents exhibit increasingly refined specificity for cells, tissues, organ structures, and physiological parameters of interest. Moreover, new optical probes can provide contrast with multiple imaging and sensing modalities, thereby increasing their potential use. New imaging modalities, measurement protocols, and instrument designs are also critical for improving the quantitative and spatial image accuracy, and utility and robustness in a wide range of biomedical settings spanning the laboratory, the operating room, and point-of-care facilities.

This feature issue of the *Journal of Biomedical Optics* on Seeing Inside Tissue with Optical Molecular Probes showcases a diverse compilation of studies pertaining to the advancement and applications of contrast agents and measurement methods. Exemplary of the depth and breadth of the field: this issue alone describes advances in contrast agents for photoacoustic, fluorescence, and X-ray modalities targeting cancer, neurons, intestinal structure, and bacteria. Novel imaging and measurement approaches are presented that rely on molecular probes to enhance tissue contrast and shed light on tissue and cellular function. These techniques are tailored to enable multimodal biological imaging and manipulation or to facilitate tissue analysis in the outpatient clinic and in challenging clinical environments such as the operating room or low-resource settings.

Kilian et al.^1^ present dual-modality contrast agents consisting of barium sulfate mixed with a near infrared II pigment to enable both X-ray and photoacoustic imaging enhancement. After characterization of multiple mixtures, the optimal pigment, a cyanine tetrafluoroborate salt, was selected and used to image the intestine *ex vivo* and *in vivo* in mice. Photoacoustic and X-ray images demonstrated successful colocalization of the contrast agent enabling future pre-clinical small animal imaging using both modalities.

Also within the context of photoacoustic imaging, Ma et al.^2^ developed novel reversibly switchable thermochromic (ReST) contrast agents to improve the sensitivity of photoacoustic tomography by enabling suppression of non-switching background signals. The new water-soluble contrast agents were obtained by surface modification of commercially available reversibly switchable thermochromic microcapsules with the hydrophilic polymer alginate and respond to changes in temperature. The study demonstrates the photostability of these probes during temperature cycling and their applicability for high resolution micron-scale imaging at millimeter depths, high speed imaging for real-time monitoring of the probes, and photoacoustic tomography with ∼100-micron spatial resolution at up to 13 mm imaging depth *in vitro* and *in vivo*.

In a third study focused on optical-resolution photoacoustic microscopy, Kim et al.^3^ harness endogenous vascular contrast to monitor vasoconstriction as a function of skin depth in response to corticosteroid treatment. In contrast to the skin blanching effect typically used to assess the effectiveness of corticosteroids, the high-resolution photoacoustic images provided direct and quantitative layer-by-layer assessment of changes in vascular density as a function of time after treatment. This approach holds great promise for monitoring anti-inflammatory treatment in dermatology. A recent review article by Hui et al.^4^ (although not part of this special section) summarizes the recent development of molecular photoacoustic probes for deep tissue imaging in the last few years.

Two other studies included in the special section describe novel molecular probes with potential clinical applications. Bateman et al.^5^ detail the use of cadaveric human tissues for pre-clinical validation of novel probes including the nerve-specific fluorescent probe LGW16-03 designed to highlight and avoid nerves during surgery.

Ostadhossein et al.^6^ synthesize and characterize carbon dots with different photoluminescent properties which they find to depend on the N position in the amine ring of the diaminopyridine precursor. They utilize five different carbon dots variants to construct a bacterial sensor array coupled with linear discriminant analysis and achieve 100% separation between 5 different oral bacteria.

Progress on imaging and sensing of fluorescence molecular signals is an equally important and exciting area of progress. In this issue, Khan et al.^7^ advance global heath by developing a hand-held imaging device connected to a smartphone to guide and monitor treatment of oral squamous cell carcinoma using 5-aminolevulinic acid (ALA)-induced Protoporphyrin IX (PpIX) acting both as a photosensitizer for photodynamic therapy and as a fluorescence contrast agent for tumor imaging and treatment guidance. This intra-oral device utilizes a ratiometric technique measuring PpIX fluorescence compared with autofluorescence and producing an R-value which is able to demarcate the cancerous lesions successfully pre- and post-photodynamic therapy. The ratiometric technique yielded results in excellent agreement with ultrasound. The R-value may also be utilized to differentiate between grades of cancer. Such a new generation of portable devices coupled with improved imaging contrast will have an important impact in the management of patients in resource-limited regions of Southeast Asia where the incidence of oral carcinomas is particularly high.

Kwon et al.^8^ present another ratiometric technique for rapid surgical margin assessment following prostate cancer resection. In this pre-clinical study, murine tissue is labeled with paired antibody-based probes consisting of a targeted probe to prostate-specific membrane antigen (PSMA) and an untargeted probe. The technique, Dual probe Difference Specimen Imaging (DDSI), showed substantial contrast difference between the high PSMA expressing tumors and the minimally PSMA expressing tumors. The optimized DDSI protocol requires a total of 8 min and could greatly facilitate intraoperative tumor margin assessment in the operating room by circumventing the need for frozen section analysis.

Dubois et al.^9^ present a low cost and modular microscope to image molecular probes based on Förster resonance energy transfer (FRET). Measurements of FRET efficiency using this instrument are validated using the molecular tension sensor VinculinTS. The instrument, which is significantly less complex and an order of magnitude less costly than typical FRET setups, yielded highly reproducible FRET efficiency measurements, was sensitive to changes in FRET efficiency of less than 10%, and may be combined with optical tweezers to study mechanobiology.

Altogether these works illustrate the multiple ways by which molecular probes continue to enhance imaging and sensing on different scales and guide the development of novel biomedical instruments and devices. We thank all the contributing authors for sharing their exciting research in this field. We also thank the JBO editorial and production staff for their extensive assistance in publishing this special issue.
